# The application of blended learning in mathematics teacher education: Protocol for a systematic review

**DOI:** 10.1371/journal.pone.0292244

**Published:** 2023-09-29

**Authors:** Duong Huu Tong, Bui Phuong Uyen, Lu Kim Ngan

**Affiliations:** School of Education, Can Tho University, Can Tho City, Vietnam; University of Porto, Faculty of Medicine, PORTUGAL

## Abstract

**Introduction:**

In recent decades, especially in higher education, blended learning has become the most commonly used active teaching strategy. Because of the COVID-19 pandemic, blended learning, which combines face-to-face and online components, is believed to overcome the shortcomings of conventional teaching methods, particularly in face-to-face interactions. Based on PRISMA guidelines, this study follows the protocol for a systematic review of blended learning applications in mathematics teacher education. This systematic review study aims to comprehend the potential of blended learning for various mathematical topics, the common blended learning models, and the benefits and challenges this teaching approach presents for educational stakeholders.

**Methods:**

Searches will be performed in various electronic databases, including Scopus, ScienceDirect, Taylor & Francis Online, Mendeley, Google Scholar, and ERIC. Selected studies that satisfy the inclusion criteria will document the use of various blended learning models in a range of mathematical topics as well as the advantages and disadvantages of this method of instruction. The data extraction process will be carried out independently by various authors, and the results of the data synthesis will be reported per the chosen studies, methodological considerations, and key findings.

**Discussion:**

This review will provide information about the application of blended learning and its benefits and challenges in mathematics teacher education to support educational stakeholders in mathematics teacher education.

## Introduction

The global COVID-19 pandemic has created numerous educational challenges, requiring teachers and students to restrict face-to-face instruction to comply with disease prevention regulations. Additionally, we are transferring our attention from microworlds to modeling, computer labs, and now to the relational revolution made possible by online technologies. In that situation, blended learning is regarded as a successful pedagogical strategy that meets real-world needs. Since its inception in the early 2000s, blended learning has gained widespread recognition as a powerful way to get around many of the drawbacks of conventional teaching techniques [1], becoming a teaching trend that is applied more and more at the university level, in particular [2]. Nevertheless, until the COVID-19 pandemic, blended learning did not take off among educators or become a widespread trend.

In mathematics education, how the internet can be used in a blended learning environment characterizes collaborative learning using technology, which introduces online courses and new research problems [3]. A key factor in ensuring the caliber of mathematics instruction in general education is the preparation of mathematics teachers at the undergraduate and graduate levels. The effectiveness of instruction and teachers’ confidence is directly impacted by providing math teachers with adequate theoretical and practical knowledge. Therefore, many universities have used blended learning to train mathematics teachers because it offers continuous, flexible, and reasonable time learning methods.

The advancement of information technology and practical requirements has stimulated research on applying blended learning in education. As a result, many studies have been conducted in various settings, from primary schools to universities, with different objectives, research methodologies, and findings. In mathematics teacher education, blended learning has been reported to have various benefits and pose different stakeholder challenges. The results of [4–6] indicate that blended learning has a positive impact on the development of pre-service teachers’ TPACK skills, higher-order thinking, independent learning and research skills, collaboration and communication skills, as well as enhances pre-service teachers’ learning attitude and engagement, self-efficacy and technology integration beliefs. On the other hand, implementing blended learning in teacher education poses proper challenges to educators and students, including infrastructure and technical problems, resources and course content quality, lesson preparation time, and, more importantly, educators’ professional development and ICT skills [4–6].

It can be said that much research has been done on the application of blended learning in the education of math teachers. Given the abundance of research on blended learning in mathematics teacher education that has been published, it is appropriate to conduct a systematic review of the literature because the findings of the various pertinent studies vary widely [7]. Numerous systematic reviews have been conducted on blended learning [1, 8–13]. The systematic review [1] synthesizes five different blended learning models used in introductory programming courses and discusses the effects of each of these models on the learning process of beginning programmers. Study [8] conducts a systematic review of systematic reviews on blended learning to identify its trends, gaps and future directions. The findings highlight that blended learning was mostly investigated in higher education, and most of the research is coming from developed countries, which calls for collaborations to facilitate blended learning adoption in developing countries. Besides, a lack of ICT skills and infrastructure is the challenge teachers, students and institutions encounter. According to a systematic review of the impacts of blended learning on academic performance and attitudes at teacher education programs in Turkey, [9] the majority of the studies confirmed that it has a positive impact on achieving course outcomes and helps pre-service teachers develop positive attitudes toward the courses by offering a variety of materials, receiving quick feedback, and monitoring progress. Some pre-service teachers are reportedly anxious about using ICT because of slow internet access, connection problems, etc. In most instances, implementing blended learning improved students’ knowledge of and attitudes toward mathematical content and discipline, according to a systematic review that focused on empirical experiences in mathematics education in higher education published in Web of Sciences and Scopus [12]. Additionally, blended learning’s benefits in terms of improved cognitive, affective, and soft skills have been documented in [10], as well as increased student performance and other challenges to the design of blended learning in [11, 13].

However, a systematic review study evaluating blended learning models appropriate for use in mathematics teacher education and the advantages and difficulties in implementing this teaching approach in mathematics teacher education has not yet been conducted.

For these reasons, conducting a thorough review of blended learning in training mathematics teachers at the university level is essential. This systematic review study will investigate the benefits and challenges this teaching approach poses to educators and mathematics pre-service teachers.

## Methods and analysis

We developed this systematic review protocol using the PRISMA statement (S1 Checklist).

### Ethics aspects and plans for dissemination

With the design of this study, approval by the ethics committee was not necessary.

### Research objectives

This systematic review aims to understand the application of blended learning in different mathematical topics, the common blended learning models, and the advantages and challenges this teaching approach presents for educational stakeholders in mathematics teacher education.

This review will address the main research question: What are the main advantages and challenges of the application of blended learning in mathematics teacher education?

### Search strategy

Following the PRISMA checklist, the search strategy will be implemented to increase methodological transparency and enhance the reproducibility of the findings. Additionally, due to using the acronym PICO (Population / Intervention / Comparison / Outcomes), to ensure a thorough search of the research sub-questions, we expanded the main question of this review: (1) What are the descriptive characteristics of the studies that made up the systematic review (such as years of publication and the nations where they were conducted, mathematical topics and the common blended learning models used)?; (2) What testing methods have been used to assess the effectiveness of blended learning in mathematics teacher education?; (3) What are the main advantages of blended learning in mathematics teacher education?; (4) What are the challenges associated with implementing blended learning in mathematics teacher education?

We looked for studies on implementing blended learning in mathematics teacher education using a variety of electronic databases, including Scopus, ScienceDirect, Taylor & Francis Online, Mendeley, Google Scholar, and ERIC. The databases were chosen based on their online accessibility and the breadth of educational research they provide. Nonetheless, to narrow the scope of the research, the research found in these databases was examined through the Scopus resource system, and only the works listed in the catalog were chosen.

Population, Intervention, Comparison and Outcomes (PICO) proposed by Kitchenham and Charters [14] was used to construct the search strategy. At first, the major search terms were derived from the research questions: (1) Population: mathematics teacher education, (2) Intervention: blended learning, (3) Comparison and Outcomes: these two dimensions were not included in the search strategy. Then, we will determine whether each database contains subject-specific header indexes and their synonyms (keywords) and related terms. The boolean operators "AND" and "OR" will be used to combine search terms. The Boolean OR incorporated synonyms, while the Boolean AND was used to link the search terms from population and intervention. The search items that will be used in some significant databases are listed in Table 1, and each database’s specific syntax requirements will require that they be modified.

**Table 1:** Search items in databases.

Databases	Search items
Scopus	TITLE-ABS-KEY (("mathematics pre-service teachers" OR "prospective mathematics teachers" OR "mathematics teacher education" OR "mathematics") AND ("blended learning" OR "blended course" OR "hybrid learning" OR "hybrid course" OR "flipped classroom"))
ScienceDirect	Title, abstract, keywords: mathematics ("blended learning" OR "blended course" OR "hybrid learning" OR "hybrid course" OR "flipped classroom") Title, abstract, keywords: mathematics pre-service teachers ("blended learning" OR "blended course" OR "hybrid learning" OR "hybrid course" OR "flipped classroom") Title, abstract, keywords: prospective mathematics teachers ("blended learning" OR "blended course" OR "hybrid learning" OR "hybrid course" OR "flipped classroom") Title, abstract, keywords: mathematics teacher education ("blended learning" OR "blended course" OR "hybrid learning" OR "hybrid course" OR "flipped classroom")
Taylor & Francis Online	[All: "mathematics"] AND [[All: "blended learning"] OR [All: "blended course"] OR [All: "hybrid learning"] OR [All: "hybrid course"] OR [All: "flipped classroom"]] [All: "pre-service mathematics teachers"] AND [[All: "blended learning"] OR [All: "blended course"] OR [All: "hybrid learning"] OR [All: "hybrid course"] OR [All: "flipped classroom"]] [All: " prospective mathematics teachers "] AND [[All: "blended learning"] OR [All: "blended course"] OR [All: "hybrid learning"] OR [All: "hybrid course"] OR [All: "flipped classroom"]] [All: " mathematics teacher education "] AND [[All: "blended learning"] OR [All: "blended course"] OR [All: "hybrid learning"] OR [All: "hybrid course"] OR [All: "flipped classroom"]]
Mendeley	(pre-service mathematics teachers) AND (blended learning); (prospective mathematics teachers) AND (blended learning); (mathematics teacher education) AND (blended learning); (pre-service mathematics teachers) AND (flipped classroom); (prospective mathematics teachers) AND (flipped classroom); (mathematics teacher education) AND (flipped classroom); (pre-service mathematics teachers) AND (hybrid learning); (prospective mathematics teachers) AND (hybrid learning); (mathematics teacher education) AND (hybrid learning)
Google Scholar	mathematics ("blended learning" OR "blended course" OR "hybrid learning" OR "hybrid course" OR "flipped classroom") mathematics pre-service teachers ("blended learning" OR "blended course" OR "hybrid learning" OR "hybrid course" OR "flipped classroom") prospective mathematics teachers ("blended learning" OR "blended course" OR "hybrid learning" OR "hybrid course" OR "flipped classroom") mathematics teacher education ("blended learning" OR "blended course" OR "hybrid learning" OR "hybrid course" OR "flipped classroom")
ERIC	mathematics ("blended learning" OR "blended course" OR "hybrid learning" OR "hybrid course" OR "flipped classroom") mathematics pre-service teachers ("blended learning" OR "blended course" OR "hybrid learning" OR "hybrid course" OR "flipped classroom") prospective mathematics teachers ("blended learning" OR "blended course" OR "hybrid learning" OR "hybrid course" OR "flipped classroom") mathematics teacher education ("blended learning" OR "blended course" OR "hybrid learning" OR "hybrid course" OR "flipped classroom")

Source: The authors, 2022.

To search for studies relevant to the objective of this study, the search terms used on the databases included ’blend learning’ + ’mathematics teacher education’, ’blend learning’ + ’pre-service mathematics teachers’, ’blended learning’ + ’prospective mathematics teachers’, ’hybrid learning’ + ’mathematics teacher education’, ’hybrid learning’ + ’pre-service mathematics teachers’, ’hybrid learning’ + ’prospective mathematics’, ’flipped classroom’ + ’mathematics teacher education’, ’flipped classroom’ + ’mathematics pre-service teachers’, and ’flipped classroom’ + ’prospective mathematics teachers’.

When searching for relevant studies, possible protocol changes will be made based on the preferred reporting items for systematic reviews [15], and the search terms may be modified to find more relevant studies.

### Selection of studies

Population, Intervention, Comparison and Outcomes (PICO) were used to construct the inclusion and exclusion criteria. Table 2 displays the criteria for inclusion and exclusion.

**Table 2:** Inclusion and exclusion criteria.

PICOS acronym	Inclusion criteria	Exclusion criteria
P—Population	Mathematics pre-service teachers	Students in other majors
I—Intervention	Examine both face-to-face and online components	Only examine face-to-face or online components
C—Control or Comparisson	-	-
O—Outcomes	Experimental or survey studies that proved the benefits and challenges of blended learning	Other types of study

Source: The authors, 2022.

The only publication types that will be covered are journal articles. Furthermore, studies published between 2012 and 2022 and written in English are acceptable. All genders will be represented in the population, including pre-service math teachers and math teacher students.

Experimental or survey studies will also examine the benefits and challenges of blended learning with both face-to-face and online components. There are limitations to including other study types, such as theoretical research and systematic reviews.

To avoid the risk of bias in including studies, two out of three members of the research team will independently search the databases for studies using specific search terms and then include or exclude studies using full-text screenings and previously established inclusion and exclusion criteria to avoid bias in the discovery of pertinent research. The third reviewer will be consulted if any disagreement or uncertainty emerges during the screening process. All researchers give their informed consent after discussing the included studies.

### Screening

After searching for relevant studies, the End-Note reference management software will be used to create the database of these articles, including titles, abstracts, and full texts. Each author will independently screen studies based on their titles and abstracts before deciding to include or exclude articles after removing duplicate articles and those not meeting the pre-specified inclusion criteria. The remaining articles will be carefully reviewed through full-article reviews to determine the definitive studies included in the systematic review. The procedure for choosing articles is outlined in a PRISMA flowchart.

### Data extraction

The full-text screening will be performed by different authors individually. Once the study selection process reaches consensus, a standard form for extracting data will be made. The information to be extracted from the selected studies includes (1) Study identification (title of article, title of journal, authors, authors’ affiliation countries and year of publication); (2) Research methods (research design, study objectives, participants and sample size, groups and controls, intervention duration, assessment instruments); (3) Main findings (blended models used, benefits and challenges of blended models). All extracted data will be stored electronically using Microsoft Excel. After performing data extraction, disagreement between authors will be resolved by discussion.

Moreover, evaluating the included studies’ methodological quality is crucial in the selection process. This evaluation aims to rate the methodological quality of a study and assess the degree to which bias has been taken into account in the study’s planning, execution, and analysis [16]. Utilizing a critical appraisal tool created by Rowe et al. (2012) [17], the quality assessment will be carried out, which was used effectively in the research [1], see Table 3. Because the included studies have different designs, assessing the quality of all significant research methodology components, including the theoretical framework, study design, data collection, data analysis, interpretation, and conclusions, is important. The quality appraisal made it possible to determine the relative advantages and disadvantages of the included studies [1]. All papers selected for inclusion in this systematic review will be subjected to rigorous appraisal by two critical appraisers.

**Table 3:** Methodological quality appraisal tool.

Criterion		
Background/literature review	❏ Detailed	❏ Limited
Sample	❏ Well described	❏ Poorly described
Study design or methodology	❏ Well described	❏ Poorly described
Outcome measures	❏ Well described	❏ Poorly described
Conclusions	❏ Supported by the study findings	❏ Not supported by the study findings

Source: The authors, 2022.

### Data synthesis

Studies will be grouped for synthesis based on their findings (benefits and challenges of blended learning), study design (experimental and survey studies), bias assessment risks (studies with a low bias risk), and studies that answer the review question [18].

Following the themes that emerged from the research question, a results report for the data synthesis will be produced based on the selected studies’ identification, procedures, and significant findings. Firstly, study identification will be examined. The data will be arranged into tables or graphically represented, with the distribution of publication years and the affiliation countries of the authors specified. Secondly, the methodological features of the chosen studies will be looked into, including research design, the blended model applied to teaching various mathematics subtopics, and evaluation tools. Finally, the chosen studies’ results will be synthesized to show the advantages and challenges of using blended learning in mathematics teacher education.

As mentioned in [19], heterogeneity will be tested using the I^2^ statistic, which can quantify heterogeneity ranging from 0% (no heterogeneity) to 100%. Additionally, three authors will utilize the Grading of Recommendations Assessment, Development, and Evaluation (GRADE) system to independently grade the quality of the results’ supporting evidence [20]. There will be four categories for evidence quality: high, moderate, low, and very low.

### Patient and public involvement

No patient or public will be involved because this is a systematic review protocol.

## Discussion and conclusion

This systematic review protocol outlines each step of the systematic review process, including the methodology for choosing studies to include and extracting and synthesizing data. According to this protocol, PRISMA guidelines will be applied, the acronym PICO will be used to construct the search strategy, and the I^2^ statistic will also test for heterogeneity. At the same time, the inclusion and exclusion criteria and the methodological quality of the included studies will be evaluated through appraisal tools.

The systematic review will analyze experimental and survey studies on the application of blended learning in mathematics teacher education in 2012–2022. The study has synthesized analytical results in many aspects corresponding to the research questions. The systematic reviews will detail (1) the characteristics of the included studies, such as distribution of affiliation countries and years of publication, the use of various blended learning models in a range of mathematical topics, (2) research methodologies, (3) the advantages and (4) challenges this method of instruction presents for stakeholders of mathematics teacher education. In terms of advantages, this systematic review will assess the impact of blended learning on pre-service teachers’ academic achievement, knowledge, skills and attitudes and the effectiveness of educators’ teaching process. The positive results will demonstrate the superiority of blended learning to online learning or conventional teaching. In the case of negative results, it motivates educators to reassess how they use blended learning in their teaching design. Besides, this systematic review is expected to clarify different challenges that educators and pre-service teachers face when learning with blended learning. Common challenges reported in previous studies, including infrastructure and technical problems, the lack of ICT skills and technology beliefs of educators and pre-service teachers, are revealed to pose threats to reap its advantages. The findings of the systematic review studies of this study can help create a roadmap about blended learning, offer stakeholders an overview of the research on blended learning applications in mathematics teacher education to facilitate blended learning adoption in their teaching and learning, and can be used as a guide for future studies on blended learning.

This protocol can be considered as a reference for future systematic reviews about blended learning. However, this protocol and the proposed systematic review certainly have limitations that need to be considered by researchers when conducting research. For instance, the protocol indicates that the selection of studies will be limited to certain databases, and only experimental and survey studies will be included. Although it is undeniable that this search strategy will help to guarantee the quality of the included studies and the extracted data, it also means that some types of research, such as case studies, observation, and qualitative research, will be excluded. Due to this, some important findings might be missed. Nevertheless, these limitations do not seem to lessen the relevance of the protocol but recommend certain insights for future studies of the research topic. In addition, aspects that this study has not analyzed can be considered for future new research, such as (1) performing systematic reviews on an aspect specifically when applying blended learning (e.g., models, teaching designs, advantages, challenges and solutions) in other fields of education, (2) conducting studies on face-to-face components in blended learning, (3) conduct studies on the influence of blended learning on the development of specific types of competencies and skills.

## Supporting information

S1 Checklist PRISMA-P checklist. (DOCX)
